# Partially Manganese-Substituted Li-Rich Antiperovskite (Li_2_Fe)SeO Cathode for Li-Ion Batteries

**DOI:** 10.1021/acsomega.5c05612

**Published:** 2025-09-01

**Authors:** Nico Gräßler, Mohamed A.A. Mohamed, Lennart Singer, Denis Djendjur, Bowen Dong, Jonah Homm, Rasha Ghunaim, Mohammad Murar, Samuel Froeschke, Silke Hampel, Rüdiger Klingeler

**Affiliations:** † 28394 Leibniz Institute for Solid State and Materials Research (IFW) Dresden e.V, Helmholtzstraße 20, Dresden 01069, Germany; ‡ Department of Physics, Faculty of Science, Sohag University, Sohag 82524, Egypt; § Kirchhoff Institute for Physics, 226227 Heidelberg University, Heidelberg 69120, Germany; ∥ Department of Applied Chemistry and Biology, Palestine Polytechnic University, Hebron, P.O. Box 198 198, Palestine

## Abstract

Lithium-rich antiperovskites are promising high-capacity cathode materials for next-generation Li-ion batteries. In this study, partially Mn-substituted (Li_2_Fe_1–*y*_Mn_*y*_)­SeO (*y* = 0.1–0.4) cathode materials were synthesized via solid-state reaction. Structural analysis confirmed the cubic antiperovskite phase with lattice expansion proportional to the Mn content, while thermal analysis revealed congruent melting behavior with improved thermal stability. Electrochemical characterization demonstrated good cycling stability across all Mn-substituted compositions. The (Li_2_Fe_0.9_Mn_0.1_)­SeO cathode exhibited an initial discharge capacity of 123 mAh/g and excellent cycling stability, but higher Mn content led to a performance decline. Battery optimization for (Li_2_Fe_0.9_Mn_0.1_)­SeO, including electrolyte selection and electrode formulation, had a pronounced impact on performance. Notably, increasing the active material ratio from 70 to 85 wt % in the electrode mixture resulted in a significantly enhanced discharge capacity, achieving 203 mAh/g in the second cycle and maintaining 85% of this value (173 mAh/g) after 100 cycles. These results underscore the importance of composition and electrode design in maximizing the electrochemical performance of Li-rich antiperovskites.

## Graphical abstract

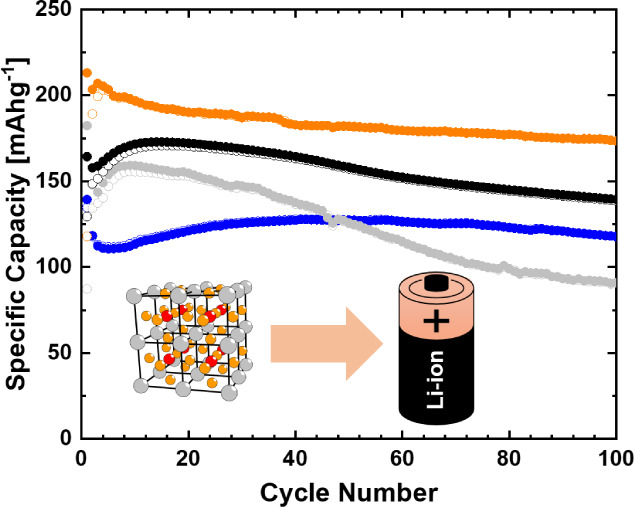

## 1 Introduction

Li-rich antiperovskites with the general formula (Li_2_ *TM*)*Ch*O (*TM* = Fe, Co, Mn; *Ch* = S, Se) represent a promising class of cathode materials for both conventional Li-ion and emerging all-solid-state batteries.^1–7^ These materials offer several advantages, including cost-effectiveness, the use of abundant and environmentally benign raw materials, efficient lithium-ion diffusion, and multielectron storage. As illustrated in Figure 1, these compounds crystallize in a cubic antiperovskite crystal structure (space group: *Pm3̅̅3m*), distinguished by the random distribution of Li^+^ and TM^2+^ ions at the same atomic position (*3c*).^8,9^ This intrinsic cationic site disorder is a defining feature of antiperovskites, significantly influencing their electrochemical behavior.^10–12^ Moreover, the structural flexibility afforded by cationic and/or anionic substitution provides opportunities to optimize chemical stability, operating voltage, and specific capacity.

**Figure 1:**
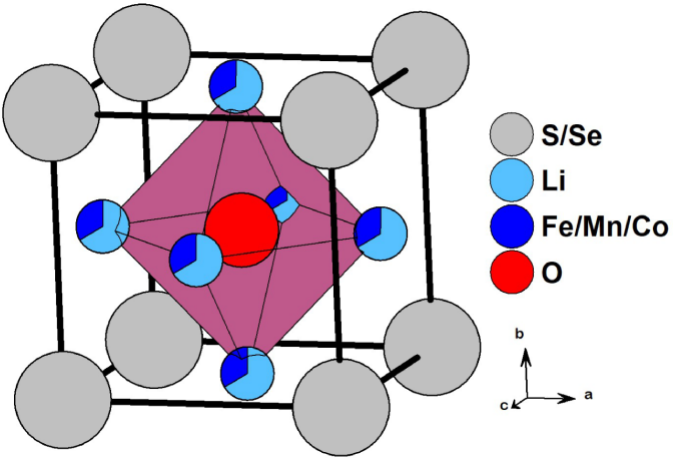
Illustration of the crystal structure of Li-rich antiperovskite cathodes.

Prior investigations have established the significant promise of Li-rich antiperovskites as cathode materials.^13–19^ For instance, (Li_2_Fe)­SeO delivers specific capacities of 150 mAh/g at a C/10 current rate and 100 mAh/g at 1 C, while exhibiting excellent cycling stability.^13^ Its sulfur-based analogue (Li_2_Fe)­SO achieves much higher capacities of 200–400 mAh/g at C/10 and 200 mAh/g at 1 C, although often accompanied by challenges in cycling stability.^15,16,18^ Limiting the potential window to avoid the partially irreversible high-voltage anionic redox process in (Li_2_Fe)­SO significantly improves the cycling stability, enabling capacities exceeding 175 mAh/g over 100 cycles at 1 C with minimal capacity fading (<4%).^15^ Deng *et al.* reported an improved electrochemical performance of (Li_2_Fe)­SO by partial substitution of Fe with Mn.^11^ While higher Mn content (e.g., (Li_2_Fe_0.5_Mn_0.5_)­SO) deteriorates the performance, moderate Mn substitution improves the cycling stability and capacity. For instance, (Li_2_Fe_0.8_Mn_0.2_)­SO delivered a reversible capacity of 220 mAh/g at 0.1 C and retained 143 mAh/g after 100 cycles at 0.5 C, with a capacity retention of 86.6%. These findings highlight the adaptability of Li-rich antiperovskites and their potential for performance enhancements through targeted substitutions.

In this study, we systematically investigate the impact of partial substitution of Fe with Mn in Li-rich antiperovskites (Li_2_Fe_1–*y*_Mn_*y*_)­SeO (*y* = 0.1–0.4). Among the synthesized materials, (Li_2_Fe_0.9_Mn_0.1_)­SeO exhibited the most promising performance, which could be further improved by optimizing battery parameters to enhance long-term cycling stability and delivered capacity.

## 2 Experimental Section

The compounds (Li_2_Fe_1–*y*_Mn_*y*_)­SeO (with *y* = 0.1–0.4) were synthesized via a solid-state reaction using stoichiometric amounts of Li_2_O (Alfa Aesar, 99.5%), elemental selenium (Alfa Aesar, 99.5%), and the transition metals Fe and Mn (both from Alfa Aesar, >99.9%). The synthesis followed established protocols as described in previous studies.^14,17^ In an Ar-filled glovebox (MBraun, Germany) with O_2_ and H_2_O concentration below 1 ppm, the weighed powders were thoroughly mixed in an agate mortar and loaded into a corundum crucible, which was subsequently placed inside a silica tube. To reduce air exposure during transfer of the silica tube to the gas burner for melt-sealing, the silica tube was temporarily closed by a rubber stopper inside the glovebox. Before sealing, the silica tube is evacuated and refilled with argon three times, and the final pressure is maintained at 0.2 bar. The ampule was then heated in a furnace to 750 °C at a heating rate of 50 °C/h, held at this temperature for 3 h, and subsequently quenched to room temperature. Afterward, the ampule was opened inside the glovebox, and the resulting product was collected for further characterization and measurements.

Powder X-ray diffraction measurements were performed by using a STOE STADI P diffractometer in Debye–Scherrer geometry with Mo Kα_1_ radiation (λ = 0.70926 Å) and a Mythen 1K detector (Dectris). To protect the samples from any reactions with air, the samples were loaded into glass capillaries (Mark tubes, Glass No. 14, Hilgenberg) inside the glovebox and subsequently melt-sealed outside. Inductively coupled plasma-optical emission spectroscopy (ICP-OES) (iCAP 6500 Duo View, Fa. Thermo Fisher Scientific GmbH) was used to estimate the stoichiometry of the produced compositions in terms of the molar ratios of the elements. Scanning electron microscopy (Nova NanoSEM 200) coupled with energy-dispersive spectroscopy (EDS Genesis with 15 kV accelerating voltage) was used to investigate the morphology and composition of the studied compounds. Differential thermal analysis (DTA) up to 1200 °C was performed by Setaram DTA92-2400 (alumina container) under a helium atmosphere (heating rate: 10 °C/min).

Electrochemical measurements were conducted using a VMP3 potentiostat (BioLogic) at 25 °C. Electrode preparation and cell assembly were performed in an argon-filled glovebox with controlled humidity and oxygen concentration. The electrodes were fabricated using a slurry process,^20^ wherein the active material (antiperovskite compound) was mixed with carbon black (TIMCAL, SUPER C65) and polyvinylidene fluoride (PVDF, Sigma) in a weight ratio of 70:15:15, using *N*-methyl-2-pyrrolidone (NMP, Alfa Aesar) as the solvent. For the (Li_2_Fe_0.9_Mn_0.1_)­SeO cathode, different electrode mixtures were investigated by varying the active material, carbon black, and binder ratios, using either NMP or dry isopropanol as the solvent for the cathode slurry. Detailed compositions are provided in the relevant discussions. The resulting mixture was spread onto an aluminum mesh current collector (Ø = 10 mm and 0.125 mm thickness). The electrodes were subsequently dried overnight under vacuum, pressed, and dried again to remove residual solvent. The mass loading of the electrodes ranged from 1.2 to 3.7 mg cm^–2^.^20^ For cell assembly, glass fiber (Whatman GF/D) was used as the separator, pure lithium metal foil (Aldrich) was used as the counter electrode, and the electrolyte consisted of either 1 M LiPF6 in a 1:1 mixture of ethylene carbonate and dimethyl carbonate or 1 M LiTFSI in a 1:1 mixture of dimethoxyethane (DME) and 1,3-dioxolane (DOL).^21^ Cyclic voltammetry (CV) and galvanostatic cycling measurements with potential limitation (GCPL) were performed in coin cells.^22^ 1 C is ascribed to the insertion/extraction of 1 Li^+^ from the formula unit.

## 3 Results and Discussion

### 3.1 Structure, Morphology, and Composition

Mn-substituted Li-rich antiperovskite (Li_2_Fe_1–*y*_Mn_*y*_)­SeO with *y* = 0.1–0.4 was synthesized via a one-step solid-state reaction. The XRD patterns of the polycrystalline samples confirm the successful synthesis, with the material crystallizing in the cubic *Pm*3̅*m* space group and exhibiting high phase purity, as shown in Figure 2. Minor secondary phases, identified as Li_2_Se (space group: *Fm*3̅*m*), MnSe (space group: *Fm*3̅*m*), and LiFeO_2_ (space group: *Fm*3̅*m*), were observed. A progressive shift of diffraction peaks toward lower angles was observed with increasing Mn content, particularly evident for the (111) peak (Figure 2b). This angular shift was accompanied by a corresponding increase in the lattice parameter (*a*) from 4.0101 Å for *y* = 0.1 to 4.0201 Å for *y* = 0.4. This linear trend is attributed to the larger ionic radius of Mn^2+^ (0.83 Å) compared to Fe^2+^ (0.78 Å),^23^ in agreement with Vegard’s law,^24^ and indicates the formation of a homogeneous solid solution (see Figure S1).

**Figure 2:**
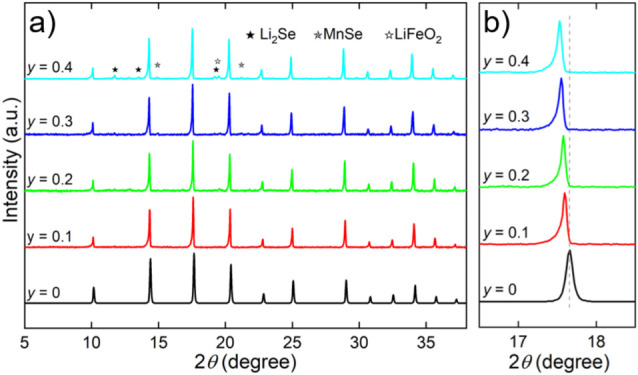
(a) XRD patterns of (Li_2_Fe_1–*y*_Mn_*y*_)­SeO with *y* = 0.1–0.4 and the theoretical pattern of (Li_2_Fe)­SeO (ICSD No. 253937) as a reference and (b) the shift in angular position for the (111) peak. The impurity phases (Li_2_Se, MnSe, and LiFeO_2_) are labeled with different symbols.

Thermodynamic investigations using differential thermal analysis (DTA) reveal congruent melting behavior for the prepared samples, characterized by a single thermal feature corresponding to melting and resolidification processes during two repeated thermal cycles, as shown in Figure 3 and Figure S2. The melting point increases linearly with increasing Mn content, ranging from 1040 °C (*y* = 0.1) to 1046 °C (*y* = 0.4), slightly exceeding the reported melting point of pristine (Li_2_Fe)­SeO (∼1037 °C).^14^ This trend reflects the high thermal phase stability of the Li-rich antiperovskites. The absence of thermal processes corresponding to the detected impurity phases based on XRD (Li_2_Se, MnSe, LiFeO_2_) further indicates their minor content.

**Figure 3:**
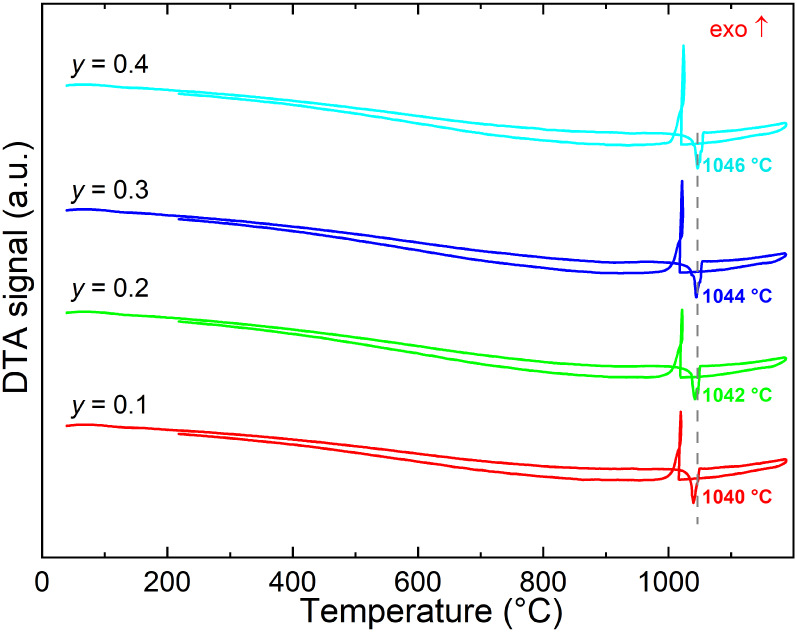
DTA thermograms of the first cycle for (Li_2_Fe_1–*y*_Mn_*y*_)­SeO (*y* = 0.1–0.4) samples with the melting point indicated.

Representative SEM images of the synthesized samples reveal no significant difference in particle size or shape with increasing Mn content (Figure 4). The (Li_2_Fe_1–*y*_Mn_*y*_)­SeO materials exhibit irregularly shaped particles with a broad size distribution, consistent with the overall morphology observed for (Li_2_Fe)­SeO prepared by the solid-state method.^14^ Particle sizes range from several tens of micrometers to submicrometer. It was observed that the surface of (Li_2_Fe_0.6_Mn_0.4_)­SeO particles appeared smoother compared with other compositions. This morphological characteristic may arise from sintering effects during synthesis, which can become more pronounced at higher Mn contents. Quantitative analysis performed via SEM-EDX confirms the presence of elemental Fe, Mn, Se, and O in varying amounts, in agreement with the theoretically expected quantities of the elements (see Figure S3). Small deviations from the ideal values are within the margin of measurement error. The homogeneity of the elemental distribution (Fe, Se, and O) within the particles was confirmed by SEM-EDS mapping images, as shown in Figure S4. However, due to the limitations of EDS, Li content was undetectable. Complementary ICP-OES measurements further corroborate the elemental ratios, with deviations from targeted stoichiometries remaining minor (Table S1). It should be noted that the presence of slight defects in the lithium content cannot be excluded. The gradual lithium loss from the structure may also be due to the sensitivity of the material to humid air.

**Figure 4:**
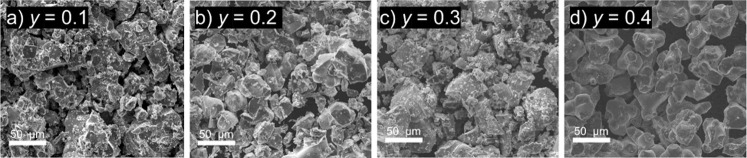
SEM images of the prepared samples: (a) (Li_2_Fe_0.9_Mn_0.1_)­SeO, (b) (Li_2_Fe_0.8_Mn_0.2_)­SeO, (c) (Li_2_Fe_0.7_Mn_0.3_)­SeO, and (d) (Li_2_Fe_0.6_Mn_0.4_)­SeO.

### 3.2 Electrochemistry

Figure 5 shows the cyclic voltammograms (CVs) of (Li_2_Fe_1–*y*_Mn_*y*_)­SeO cathodes measured at a scan rate of 0.1 mV/s. Initially, a wide potential range (1–4.5 V) was selected for (Li_2_Fe_0.9_Mn_0.1_)­SeO to evaluate its capacity for multielectron storage via both cationic and anionic redox processes. However, no significant redox activity was observed above 3 V. Therefore, subsequent investigations were confined to the potential range of 1–3 V, which is more relevant for practical applications.

**Figure 5:**
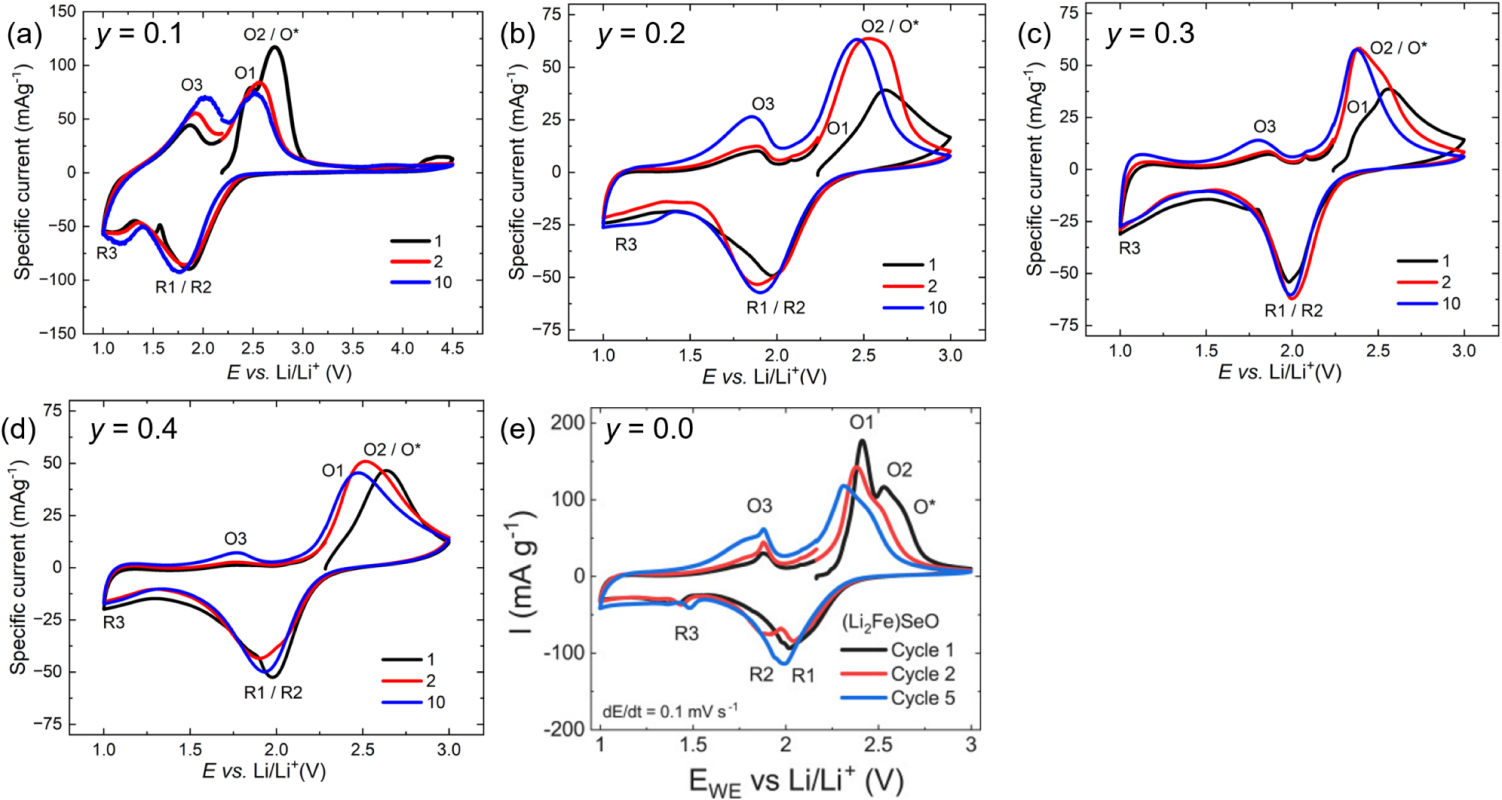
Cyclic voltammograms of the prepared cathodes at a scan rate of 0.1 mV/s: (a) (Li_2_Fe_0.9_Mn_0.1_)­SeO, (b) (Li_2_Fe_0.8_Mn_0.2_)­SeO, (c) (Li_2_Fe_0.7_Mn_0.3_)­SeO, and (d) (Li_2_Fe_0.6_Mn_0.4_)­SeO. For comparison, (e) the CV of pristine (Li_2_Fe)­SeO is reprinted with permission from ref.^14^ Copyright 2023 Elsevier. Oxidation and reduction processes (O/R) for the first cycle are marked and discussed in the text.

For comparison, the CVs of pristine (Li_2_Fe)­SeO (Figure 5e) exhibit distinct redox behavior, characterized by multistage processes. The two-step cationic redox process is marked by the oxidation peaks O1/O2 and the corresponding reduction peaks R1/R2. Additionally, a shoulder feature (O*) can be recognized, which is attributed to an anionic redox process involving the selenium sublattice.^14^

Substitution with Mn induces significant changes in the redox behavior of (Li_2_Fe_1–*y*_Mn_*y*_)­SeO. The CV curves display increasing broadening and asymmetry with the Mn content. These features suggest the formation of a continuous solid solution and/or the presence of multiple overlapping redox reactions. Such overlap between cationic and anionic redox contributions complicates the clear identification of individual redox peaks. A similar trend is observed in the Mn-substituted sulfide analogues: while (Li_2_Fe)­SO exhibits well-defined redox features, these become significantly broadened and less distinguishable upon Mn substitution in (Li_2_Fe_0.9_Mn_0.2_)­SO and (Li_2_Fe_0.5_Mn_0.5_)­SO.^11^

Given the structural and electrochemical similarities, the same labeling convention is applied here for (Li_2_Fe_1–*y*_Mn_*y*_)­SeO as previously used for (Li_2_Fe)­SeO. In accordance with the electrochemical standard potentials and earlier studies on oxide-based cathodes,^25–27^ Mn^2+/3+^ redox activity is expected at higher potentials than Fe^2+/3+^, as also reported in prior literature.^11,17^ However, due to overlapping redox potentials, the individual contributions from Fe and Mn are not clearly distinguishable in the CVs. It can be assumed that the extraction of lithium ions in the voltage regime between the open-circuit voltage (OCV) and 3 V is accompanied by a multistage oxidation characterized as cationic (O1 and O2, corresponding to both Fe^2+/3+^ and Mn^2+/3+^) and anionic (O*, associated with Se^2‑/*n*–^, *n* < 2) processes.^28,29^ Upon Li^+^ insertion (discharge from 3 to 1 V), broader reduction peaks (R1, R2, R3) are observed in the CV. The overlapping R1/R2 peaks correspond to the cationic redox processes, while R3 is associated with the reduction of electrochemically active transition metal selenides, likely formed due to partial structural decomposition accompanying the anionic redox process (O*).^13^ The degree of reversibility of the anionic redox activity in Li-rich antiperovskite cathodes remains uncertain and requires further investigation. Subsequent charging from 1 V to the OCV completes the electrochemical cycle, yielding an additional oxidation peak, the oxidation peak O3, corresponding to a reversible oxidation of the R3 process. The progressive broadening and gradual shift of redox peaks toward lower potentials with cycling indicate kinetic limitations and changes in the reaction mechanism, potentially due to structural rearrangements or increased resistance at the electrode–electrolyte interface, as previously reported.^3,11,13,17^ Overall, the electrochemical redox processes, including the R3/O3 process, are more pronounced in compositions with a lower Mn content.

The electrochemical performance of (Li_2_Fe_1–*y*_Mn_*y*_)­SeO was evaluated through galvanostatic cycling, as shown in Figure 6a, with selected potential profiles corresponding to differential capacity (d*Q*/d*V*) plots provided in Figure S5. Initial discharge capacities systematically decrease with increasing Mn content. In the second cycle, (Li_2_Fe_0.9_Mn_0.1_)­SeO delivers a discharge capacity of 123 mAh/g, which decreases to 102 mAh/g for (Li_2_Fe_0.8_Mn_0.2_)­SeO, 82 mAh/g for (Li_2_Fe_0.7_Mn_0.3_)­SeO, and 67 mAh/g for (Li_2_Fe_0.6_Mn_0.4_)­SeO. The reduced capacity observed in Mn-rich compositions can be primarily attributed to the diminished contribution from both cationic and anionic redox processes, as indicated by the d*Q*/d*V* plots from cycle 5 (see Figure S5). For (Li_2_Fe_0.9_Mn_0.1_)­SeO, the d*Q*/d*V* curve exhibits a broad peak centered at 2.27 V, accompanied by a pronounced shoulder extending toward higher potentials. In contrast, this peak decreases significantly in magnitude and shifts slightly to around 2.35 V for (Li_2_Fe_0.6_Mn_0.4_)­SeO. Furthermore, a substantial capacity contribution in the 1.0–2.0 V range is observed in (Li_2_Fe_0.9_Mn_0.1_)­SeO, but it becomes markedly diminished with increasing Mn substitution. This trend may indicate either a reduced participation of the R_3_/O_3_ redox couple in this potential region or a shift of these redox processes beyond the operational voltage window. Further studies are required to elucidate the underlying cause of this observation.

**Figure 6:**
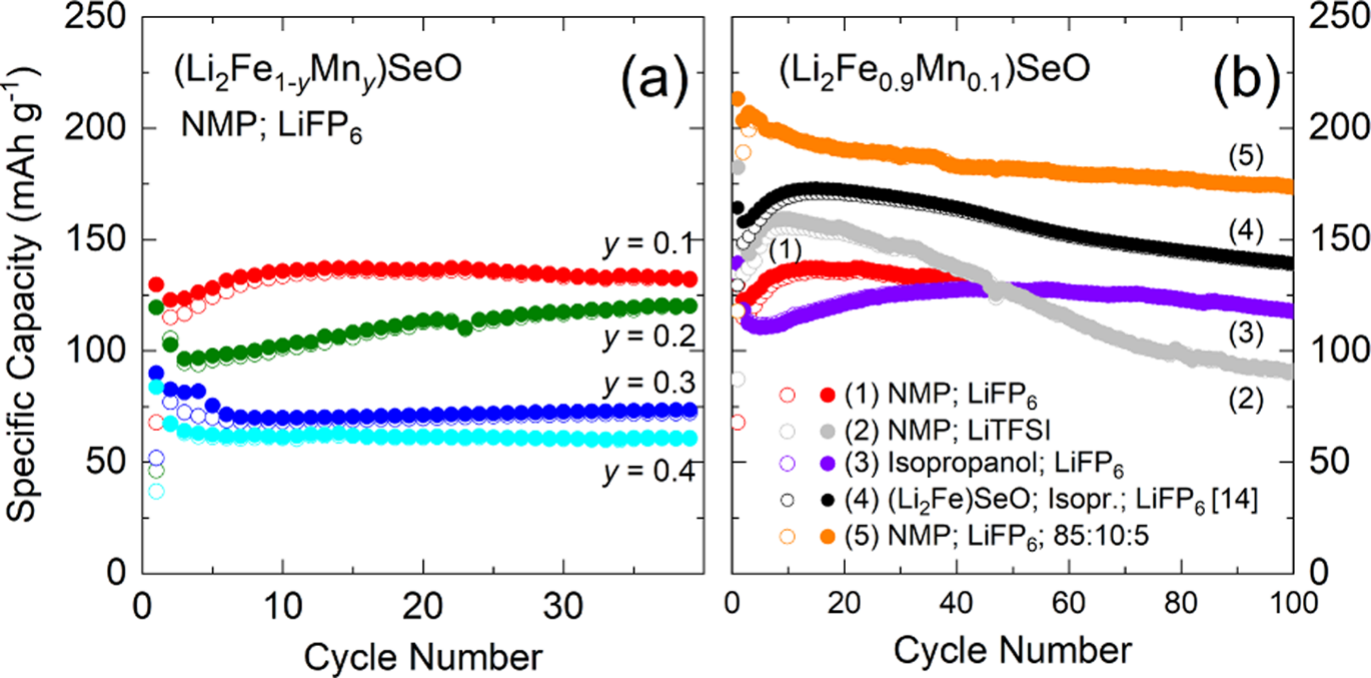
(a) Specific dis-/charge capacities at C/10 for (Li_2_Fe_1–*y*_Mn_*y*_)­SeO cathodes using LiPF_6_ as an electrolyte and NMP as a solvent for the cathode slurry. (b) Long-term cycling performance of the (Li_2_Fe_0.9_Mn_0.1_)­SeO cathode with varied electrode preparation parameters. With the exception of electrode (5), the mass ratio of active material:carbon black:binder was kept at 70:15:15. For comparison, the reported results for a (Li_2_Fe)­SeO cathode (using isopropanol as the solvent for the cathode slurry) are included, reprinted in part with permission from ref.^14^ Copyright 2023 Elsevier. Open/full markers indicate de/lithiation.

All Mn-substituted antiperovskite materials exhibit very good cycling stability over 40 cycles, with capacities either remaining constant or increasing after a few initial cycles. Specifically, (Li_2_Fe_0.9_Mn_0.1_)­SeO and (Li_2_Fe_0.8_Mn_0.2_)­SeO show gradual capacity rise, reaching discharge capacities of 132 mAh/g and 116 mAh/g at the 40th cycle, respectively.

The overall decline in electrochemical performance with higher Mn content is most likely due to the intrinsically lower redox activity of Mn^2+^ compared to Fe^2+^.^17^ This effect is associated with the Jahn–Teller distortion of Mn^3+^ (high-spin, *d* ^4^) which induces structural distortions in the crystal lattice that may impede lithium-ion diffusion and hinder the reversibility of the Mn^2+/3+^ redox couple. Similar behavior has been reported in other Mn-based cathode materials, such as LiMn_2_O_4_ and LiMn_1–*x*_Fe_*x*_PO_4_.^30–32^

Given the promising performance of (Li_2_Fe_0.9_Mn_0.1_)­SeO, a systematic investigation of the experimental conditions was undertaken to further enhance its electrochemical properties. This involved varying the electrolyte composition, the solvent used for cathode slurry preparation, and the electrode formulation, while keeping all other parameters constant. For comparative purposes, the long-term cycling performance of the unsubstituted (Li_2_Fe)­SeO cathode,^14^ synthesized under similar conditions and using isopropanol as the cathode slurry solvent, is also presented (Figure 6b, black curve). The pristine (Li_2_Fe)­SeO material outperformed the Mn-substituted sample when tested under identical conditions (70:15:15 electrode ratio, LiPF_6_ electrolyte, and isopropyl alcohol as the slurry solvent).

The influence of electrolyte composition was assessed by using LiTFSI in DOL/DME. This electrolyte resulted in an initial capacity of around 148 mAh/g, which is comparable to that observed with LiPF_6_. However, significant capacity fading occurred, with retention dropping to 61% (90 mAh/g) after 100 cycles. A similar trend of pronounced capacity degradation was previously reported for the sulfur-containing material (Li_2_Fe_0.5_Mn_0.5_)­SO.^17^

In contrast, the utilization of 2-propanol as the solvent for the cathode slurry resulted in enhanced cycling stability for the resulting electrode. The so-prepared electrode exhibits a maximum of ∼ 127 mAh/g at cycle 56. After this point, a slight capacity fade is observed, resulting in ∼118 mAh/g after 100 cycles, which is still ∼5% higher than the initial reversible capacity at cycle 3. Compared to the electrode prepared using NMP as a cathode slurry solvent, the capacity is slightly lower during the first 40 cycles. The observed behavior may be related to the differing effects of NMP and isopropyl alcohol on the electrode microstructure. In particular, the higher ability of NMP to dissolve the PVDF binder compared to isopropanol can lead to better binder distribution combined with higher utilization of the active materials, resulting in the higher initial capacity^33^

A significant improvement in the electrochemical performance was achieved by varying the electrode composition. Increasing the active material ratio to 85:10:5 wt % (active material:carbon black:binder) resulted in an impressive initial capacity of 203 mAh/g. However, this formulation entailed a slight decrease in capacity during cycling, and the electrode still retained 85% of its initial capacity (173 mAh/g) after 100 cycles. The potential profile (see Figure S6) suggests that the enhanced capacity is attributed to the increased electrochemical activity of both cationic and anionic redox processes. Additionally, a significant contribution to the reversible discharge capacity below ∼1.3 V is likely associated with an Fe_1–*x*_S_*x*_ phase.^15^

Notably, the low requirement for conductive additives indicates the high intrinsic conductivity of the Li-rich antiperovskite material, as supported by the all-solid-state battery study.^7^

This study demonstrates that while pristine (Li_2_Fe)­SeO exhibits superior initial performance compared with Mn-substituted analogues under standard conditions, targeted electrode optimization can significantly enhance the electrochemical properties of the Mn-substituted material. Specifically, optimized formulations of (Li_2_Fe_0.9_Mn_0.1_)­SeO achieved comparable or superior long-term performance, underscoring the importance of electrode architecture and processing parameters. The investigated parameters are summarized in Table 1, offering practical guidance for future development of Li-rich antiperovskite cathodes.

**Table 1:** Comparative Electrochemical Performance of (Li_2_Fe_1–*y*_Mn_*y*_)­SeO Cathodes

composition	electrode formulation (active material:carbonblack:binder) (wt.%)	electrolyte	slurry solvent	discharge capacity after 2/40/100 cycles (mAh/g)
(Li_2_Fe_0.9_Mn_0.1_)SeO	70:15:15	LiPF_6_	NMP	123/132/-
(Li_2_Fe_0.8_Mn_0.2_)SeO	70:15:15	LiPF_6_	NMP	102/116/-
(Li_2_Fe_0.7_Mn_0.3_)SeO	70:15:15	LiPF_6_	NMP	82/73/-
(Li_2_Fe_0.6_Mn_0.4_)SeO	70:15:15	LiPF_6_	NMP	67/61/-
(Li_2_Fe_0.9_Mn_0.1_)SeO	70:15:15	LiTFSI	NMP	147/137/90
(Li_2_Fe_0.9_Mn_0.1_)SeO	70:15:15	LiPF_6_	Isopropanol	118/127/118
(Li_2_Fe)SeO^14^	70:15:15	LiPF_6_	Isopropanol	158/164/139
**(Li**_**2**_ **Fe**_**0.9**_ **Mn**_**0.1**_ **)SeO** **(optimized)**	**85:10:5**	**LiPF**_**6**_	**NMP**	**203/183/173**

Overall, the results clearly show that both the intrinsic elemental composition and the extrinsic electrode fabrication process critically influence the electrochemical behavior. Future studies should focus on systematically investigating these variables, particularly the role of slurry solvent, the active material:carbon:binder ratio, and electrolyte formulation, to establish best practices for maximizing performance and cycling stability in this emerging class of cathode materials.^34–36^

## 4 Conclusions

Partially Mn-substituted Li-rich antiperovskites (Li_2_Fe_1–*y*_Mn_*y*_)­SeO (*y* = 0.1–0.4) were successfully synthesized via solid-state reaction, exhibiting cubic structures and lattice expansion proportional to Mn content. The thermal stability of the compounds increased linearly with Mn substitution. All investigated Mn-substituted Li-rich antiperovskite cathodes demonstrate good cycling stability, retaining over 90% of their capacity after 40 cycles. However, initial discharge capacities systematically decrease with increasing Mn content, from 123 mAh/g for (Li_2_Fe_0.9_Mn_0.1_)­SeO to 67 mAh/g for (Li_2_Fe_0.6_Mn_0.4_)­SeO. This capacity reduction is attributed to the diminished redox activity of both cationic and anionic processes, likely due to the Jahn–Teller effect of Mn^3+^. Further investigations on the (Li_2_Fe_0.9_Mn_0.1_)­SeO cathode by varying the electrolyte composition, solvent for cathode slurry, and electrode formulation show significant influence on long-term cycling performance. Notably, increasing the active material content from 70 wt % to 85 wt % in the electrode mixture results in an impressive initial capacity of 203 mAh/g, with 85% retention after 100 cycles. Future research should focus on strategies to maximize energy density while maintaining long-term cycling stability. This includes pursuing advanced structural characterization techniques,^11,37–40^ such as *operando* XRD, X-ray absorption spectroscopy (XAS), and pair distribution function (PDF) analysis, to fully elucidate the complex redox mechanisms and structural evolution during cycling, thereby guiding the rational design of next-generation Li-rich antiperovskite cathodes.

## Supplementary Material



## Notes

Lattice parameter; DTA thermograms; EDS spectra; SEM images with EDS element mapping; ICP-OES data; potential profiles with d*Q*/d*V* plots (PDF)
