# Supplementary material for: Partially Manganese-Substituted Li-Rich Antiperovskite (Li2Fe)SeO Cathode for Li-Ion Batteries
Source: ACS Omega. 2025 Sep 1;10(36):41719–26. doi: 10.1021/acsomega.5c05612 (PMC12444673; doi:10.1021/acsomega.5c05612)
Supplement: Supplementary file 1 [file ao5c05612_si_001.pdf]

Supplementary Information

Partially Manganese-Substituted Li-rich Antiperovskite (Li_2Fe)SeO Cathode for Li-ion Batteries

Nico Gräßler[a,], Mohamed A.A. Mohamed[a,b], Lennart Singer[c], Denis Djendjur[c], Bowen Dong[c], Jonah Homm[c], Rasha Ghunaim[a,d], Mohammad Murar[a,d], Samuel Froeschke[a], Silke Hampel[a], Rüdiger Klingeler[c,*]*

[a] Leibniz Institute for Solid State and Materials Research (IFW) Dresden e.V., Helmholtzstraße 20, Dresden 01069, Germany

[b] Department of Physics, Faculty of Science, Sohag University, Sohag 82524, Egypt

[c] Kirchhoff Institute for Physics, Heidelberg University, Heidelberg 69120, Germany

[d] Department of Applied Chemistry and Biology, Palestine Polytechnic University, Hebron, P.O. Box 198, Palestine

** corresponding authors: n.graessler@ifw-dresden.de, klingeler@kip.uni-heidelberg.de*

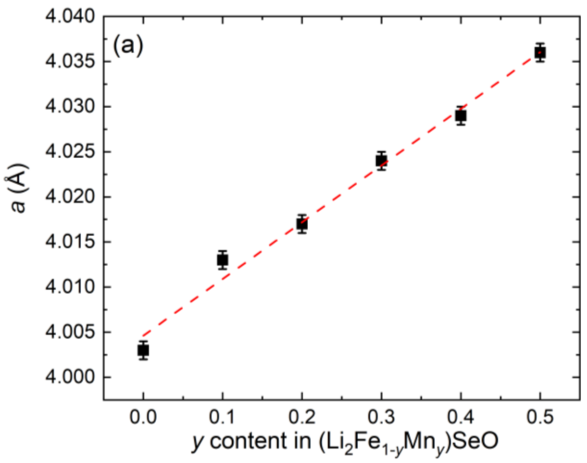

Figure S1. Variation of the lattice parameter for ($Li_2Fe_{1-y}Mn_y$)SeO with y = 0.1 - 0.4.The red dashed line is the linear fitting.

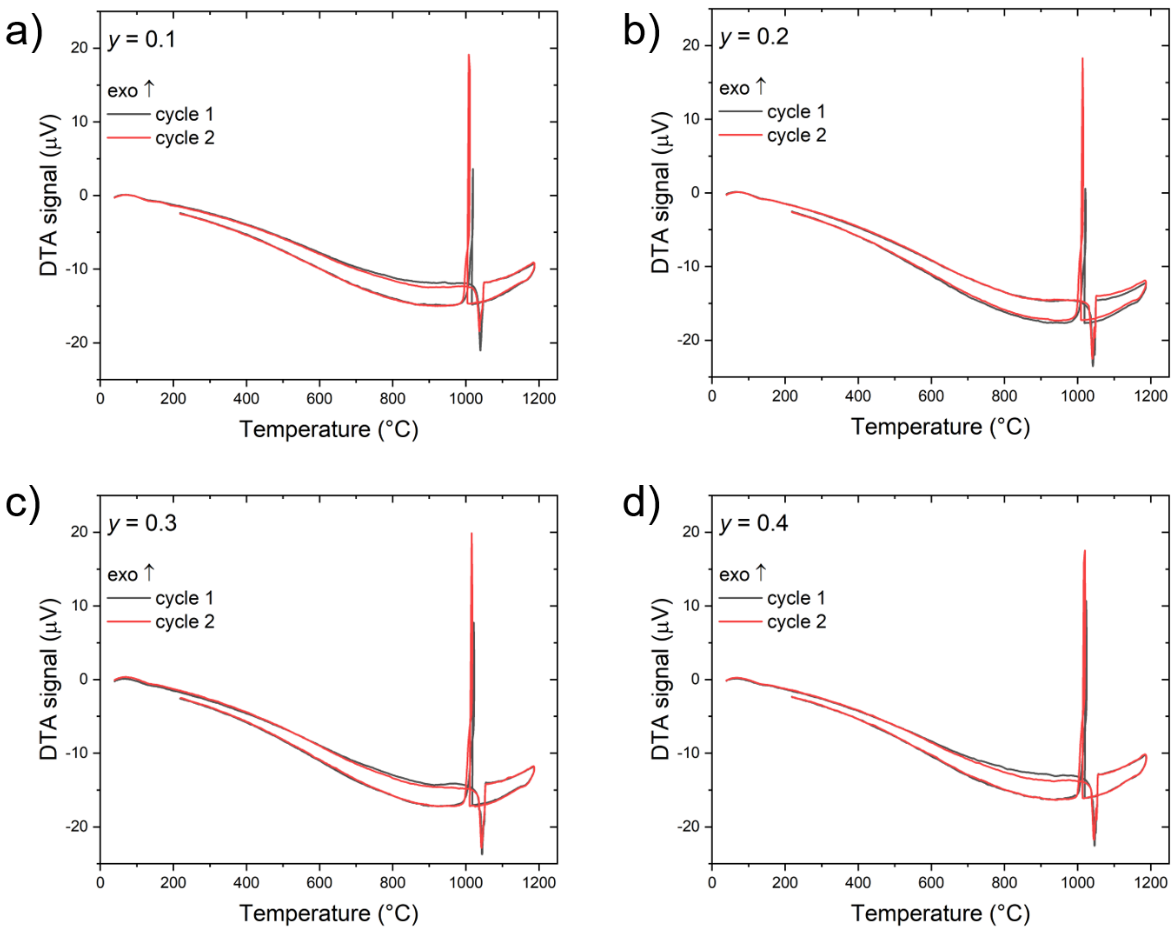

Figure S2. DTA thermograms during two thermal cycles of the prepared samples: a) $(Li_2Fe_{0.9}Mn_{0.1})SeO$, b) $(Li_2Fe_{0.8}Mn_{0.2})SeO$, c) $(Li_2Fe_{0.7}Mn_{0.3})SeO$, and d) $(Li_2Fe_{0.6}Mn_{0.4})SeO$.

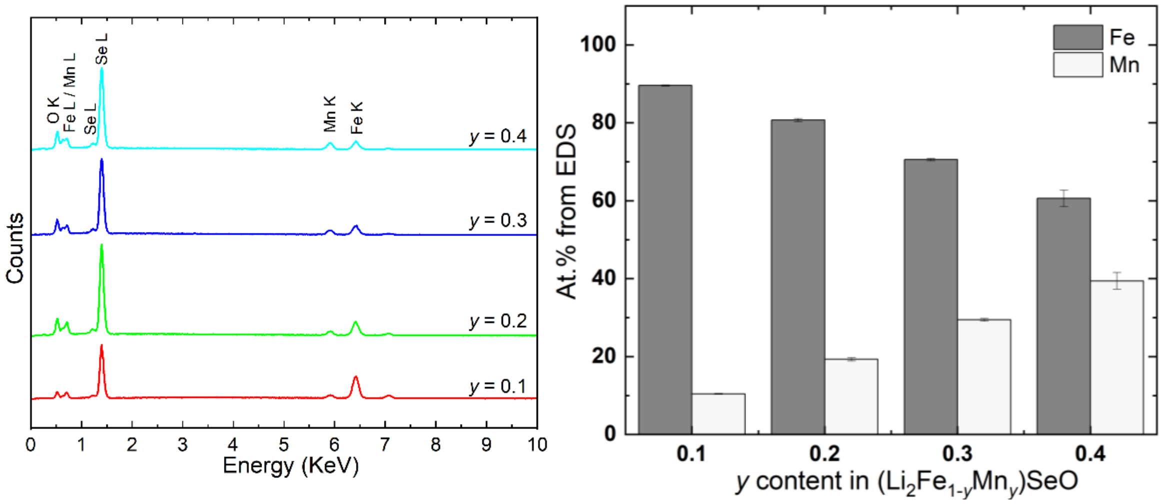

Figure S3. (left) EDS spectra for $(Li_2Fe_{1-y}Mn_y)SeO$ series and (right) variation in atomic percentages for Fe and Mn as determined by EDS.

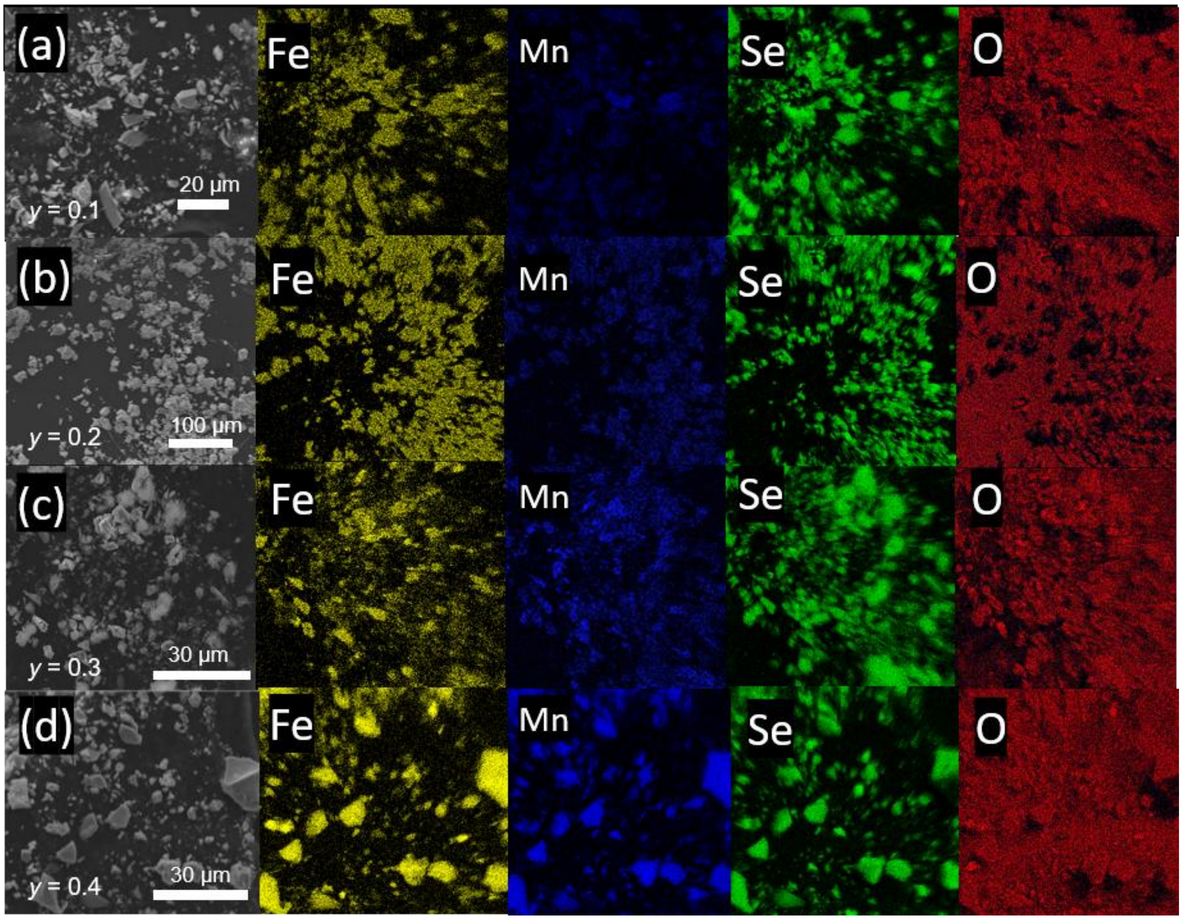

Figure S4. SEM images and corresponding EDS mapping results for a) $(Li_2Fe_{0.9}Mn_{0.1})SeO$, b) $(Li_2Fe_{0.8}Mn_{0.2})SeO$, c) $(Li_2Fe_{0.7}Mn_{0.3})SeO$, and d) $(Li_2Fe_{0.6}Mn_{0.4})SeO$.

Table S1. The obtained elemental molar ratios by ICP-OES for $(Li_2Fe_{1-y}Mn_y)SeO$ samples with y = 0.1 - 0.4. Molar ratios are scaled to sum up to about 5.

**Element**	**sample**							
	y = 0.1		y = 0.2		y = 0.3		y = 0.4	
	Mass%	Molar ratio	Mass%	Molar ratio	Mass%	Molar ratio	Mass%	Molar ratio
Li	8.23 ±0.07	1.97(7)	8.20 ±0.06	1.95(2)	8.21 ±0.05	1.98(1)	8.25 ±0.13	1.96(3)
Fe	30.11 ±0.22	0.89(1)	26.59 ±0.22	0.79(1)	23.12 ±0.18	0.69(1)	19.86 ±0.23	0.59(1)
Mn	3.46 ±0.02	0.11(1)	6.60 ±0.06	0.19(1)	10.01 ±0.06	0.31(1)	13.18 ±0.17	0.39(1)
Se	48.23 ±0.32	1.01(1)	48.50 ±0.34	1.02(1)	49.39 ±0.30	1.05(1)	48.54 ±0.41	1.01(1)
O	9.97	1.02	10.11	1.05	9.27	0.97	10.17	1.05

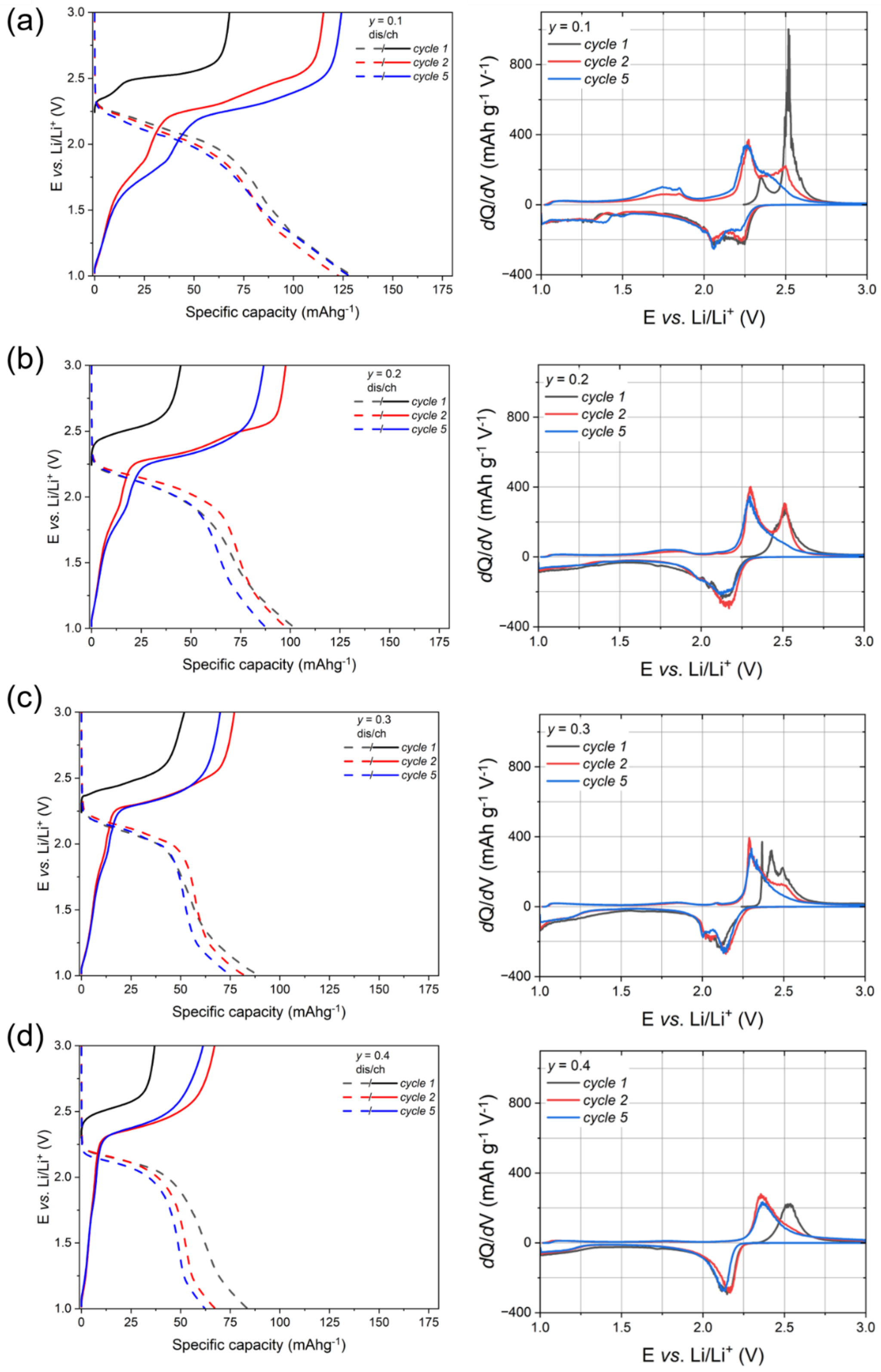

Figure S5. Selected potential profiles and corresponding dQ/dV plots of a) $(Li_2Fe_{0.9}Mn_{0.1})SeO$, b) $(Li_2Fe_{0.8}Mn_{0.2})SeO$, c) $(Li_2Fe_{0.7}Mn_{0.3})SeO$, and d) $(Li_2Fe_{0.6}Mn_{0.4})SeO$. Dashed and solid line stand for lithiation and delithiation, respectively.

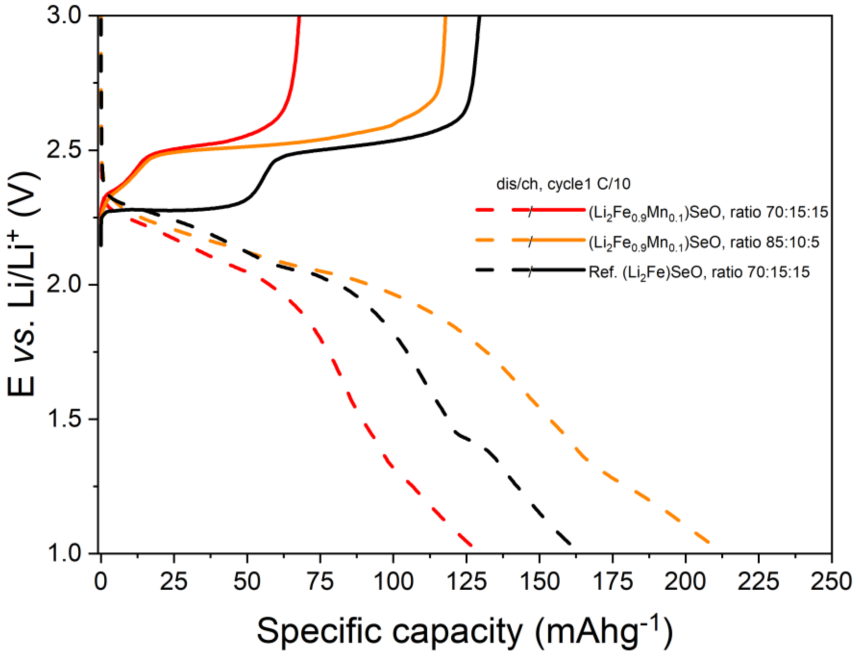

Figure S6. Comparison of the potential profiles for the cycle 1 at C/10 of ($Li_2Fe_{0.9}Mn_{0.1}$)SeO and (Li_2Fe)SeO (reprinted in part with permission from Ref [1]. Copyright 2023 Elsevier. Dashed and solid line stand for lithiation and delithiation, respectively.

The disappearance of the plateau around 2.25 V (Figure S6) with increasing Mn content is consistent with the trend observed in the *d*Q/*d*V curves (Figure S5). Rather than indicating the complete absence of Fe redox activity, this behavior can be attributed to a gradual shift of the O1 process toward higher potentials, where it begins to overlap with the O2 feature. This overlap results in a merged and broadened redox feature, making the individual contribution of Fe less distinguishable in the charge-discharge profiles.

Reference:

[1] Mohamed, M. A. A.; Singer, L.; Hahn, H.; Djendjur, D.; Özkara, A.; Thauer, E.; Gonzalez-Martinez, I. G.; Hantusch, M.; Büchner, B.; Hampel, S.; Klingeler, R.; Gräßler, N. Lithium-Rich Antiperovskite (Li_2Fe)SeO: A High-Performance Cathode Material for Lithium-Ion Batteries. *Journal of Power Sources* **2023**, *558*, 232547. https://doi.org/10.1016/j.jpowsour.2022.232547
